# Effect of Task-Correlated Physiological Fluctuations and Motion in 2D and 3D Echo-Planar Imaging in a Higher Cognitive Level fMRI Paradigm

**DOI:** 10.3389/fnins.2016.00225

**Published:** 2016-06-07

**Authors:** Jarle Ladstein, Hallvard R. Evensmoen, Asta K. Håberg, Anders Kristoffersen, Pål E. Goa

**Affiliations:** ^1^Department of Circulation and Medical Imaging, Norwegian University of Science and TechnologyTrondheim, Norway; ^2^MI Lab, Norwegian University of Science and TechnologyTrondheim, Norway; ^3^Department of Neuroscience, Norwegian University of Science and TechnologyTrondheim, Norway; ^4^Department of Radiology and Nuclear Medicine, St. Olavs University HospitalTrondheim, Norway; ^5^Department of Physics, Norwegian University of Science and TechnologyTrondheim, Norway

**Keywords:** EPI, fMRI, nuisance regressors, physiological noise, motion, BOLD

## Abstract

**Purpose:** To compare 2D and 3D echo-planar imaging (EPI) in a higher cognitive level fMRI paradigm. In particular, to study the link between the presence of task-correlated physiological fluctuations and motion and the fMRI contrast estimates from either 2D EPI or 3D EPI datasets, with and without adding nuisance regressors to the model. A signal model in the presence of partly task-correlated fluctuations is derived, and predictions for contrast estimates with and without nuisance regressors are made.

**Materials and Methods:** Thirty-one healthy volunteers were scanned using 2D EPI and 3D EPI during a virtual environmental learning paradigm. In a subgroup of 7 subjects, heart rate and respiration were logged, and the correlation with the paradigm was evaluated. FMRI analysis was performed using models with and without nuisance regressors. Differences in the mean contrast estimates were investigated by analysis-of-variance using Subject, Sequence, Day, and Run as factors. The distributions of group level contrast estimates were compared.

**Results:** Partially task-correlated fluctuations in respiration, heart rate and motion were observed. Statistically significant differences were found in the mean contrast estimates between the 2D EPI and 3D EPI when using a model without nuisance regressors. The inclusion of nuisance regressors for cardiorespiratory effects and motion reduced the difference to a statistically non-significant level. Furthermore, the contrast estimate values shifted more when including nuisance regressors for 3D EPI compared to 2D EPI.

**Conclusion:** The results are consistent with 3D EPI having a higher sensitivity to fluctuations compared to 2D EPI. In the presence partially task-correlated physiological fluctuations or motion, proper correction is necessary to get expectation correct contrast estimates when using 3D EPI. As such task-correlated physiological fluctuations or motion is difficult to avoid in paradigms exploring higher cognitive functions, 2D EPI seems to be the preferred choice for higher cognitive level fMRI paradigms.

## 1. Introduction

2D single-shot echo-planar imaging (EPI) has been the standard acquisition method in blood oxygen level dependent (BOLD) functional MRI. 2D EPI is a multi-slice method whereby each slice is individually excited and acquired one after the other, and full 2D *k*-space coverage is obtained after a single excitation. Excitation and read-out for one slice takes 50–100 ms, and the total repetition time (*T*_R_) in terms of the time interval between each time a spin population (i.e., slice) experiences an rf-pulse is approximately 2–3 s, depending on the total number of slices. For 2D EPI the spin population *T*_R_ is the same as the time required for the acquisition of one full volume (*T*_R_vol__).

3D EPI has been proposed as an alternative method, particularly for imaging at higher field strengths (Poser et al., 2010). In 3D EPI, the whole volume is excited at each rf-pulse, and a second phase-encoding direction is used for spatial encoding along the third axis, instead of using slice-selection. Therefore, in 3D EPI the spin population repetition time (*T*_R_) is equal to the rf-pulse time interval, i.e., 50–100 ms, while the time required to sample the whole 3D *k*-space (*T*_R_vol__) is *n*_3D_ times *T*_R_, where *n*_3D_ is the number of 3D phase-encoding steps.

BOLD sensitivity is usually defined as the temporal signal-to-noise ratio (tSNR) per square root of the volume repetition time (*T*_R_vol__). A number of studies have compared the tSNR and BOLD sensitivity in 3D EPI and in 2D EPI (Lai and Glover, 1998; Goerke et al., 2005; Hu and Glover, 2007; Poser et al., 2010; Lutti et al., 2013). It has been shown that 3D EPI yields comparable or higher BOLD sensitivity than 2D EPI for a range of resolutions at 7T (Poser et al., 2010), as well as at 3T for low resolution (Goerke et al., 2005) and recently for high resolution (Lutti et al., 2013). However, signal instabilities such as physiological noise behave differently and have a more adverse effect on tSNR in 3D EPI than in 2D EPI (Goerke et al., 2005; Poser et al., 2010; Kristoffersen and Goa, 2011; van der Zwaag et al., 2012; Jorge et al., 2013). This difference in vulnerability to physiological noises is linked to the differences in data acquisition schemes between 2D EPI and 3D EPI, as described above.

Methods to correct for physiological noise have been developed (Glover et al., 2000; Birn et al., 2006; Shmueli et al., 2007). Residual effects of motion are similarly corrected based on the estimated parameters from motion correction (Friston et al., 1996). It has been shown that correction of physiological noise gives a larger increase in tSNR for 3D EPI than for 2D EPI at 7 T (Jorge et al., 2013) and 3 T (Lutti et al., 2013). Overall, these studies indicate that 3D EPI could be a better choice for fMRI in many cases, particularly for high resolution imaging where the relative physiological noise contribution is lower (Triantafyllou et al., 2005). However, these previous studies used either resting state data or relatively simple fMRI paradigms.

In higher cognitive level paradigms, the active periods can result in increased heart rate and respiration, as seen for a continuous memory task (Backs and Seljos, 1994) and during simulated flying (Veltman and Gaillard, 1998). It has also been shown that heart rate and respiration can increase with greater working memory load (Backs and Seljos, 1994; Gianaros et al., 2004; Mehler et al., 2009). For simple paradigms, such as visual checkerboard, such increases in heart rate or respiration do not occur (Conrad and Klingelhöfer, 1989). This indicates that higher cognitive level paradigms can lead to changes both in respiration and cardiac pulsation. Further, it has been demonstrated that such changes in respiration and cardiac pulsation can be correlated with BOLD signal changes. For a verbal working memory task compared to perceptual-motor control task, regional cerebral blood flow correlated with heart period and high-frequency heart period variability (Gianaros et al., 2004). In a study investigating respiratory effects, it was found that increased BOLD signal for a lexical decision task, compared to rest, correlated with breathing changes throughout the brain. Adding nuisance regressors to the model to remove task-correlated breathing changes improved the fMRI data (Birn et al., 2009). Taken together, higher cognitive level paradigms, as compared to visual checkerboard or rest, can result in task-correlated signal fluctuations due to cardiorespiratory increases in the active periods.

The aim of this study was to compare high resolution BOLD fMRI acquired at 3T using 2D EPI and 3D EPI for a higher cognitive level fMRI paradigm, i.e., free exploration and learning of virtual environments. Based on a signal model derived in the theory section below, we hypothesized that the fMRI contrast estimates will be biased if the acquisition method is sensitive to partially correlated physiological fluctuations and motion. We further hypothesized that adding nuisance regressors to account for physiological fluctuations and motion, the bias in the contrast estimates will be removed and in addition the residuals should be reduced.

## 2. Theory

The relative signal change, Δ*S*∕*S*, due to the BOLD response for some task, can be expressed by Wald (2012):

(1) $$\begin{array}{l} \Delta S∕S=1-\exp\left(-T_{E}\Delta R_{2}^{*}\right)\approx -T_{E}\Delta R_{2}^{*} \end{array}$$

Here the difference in transverse relaxation times, $T_{2}^{*}$, between rest and the task has been replaced by the difference in relaxation rates $\Delta R_{2}^{*}$, with $R_{2}^{*}=1∕T_{2}^{*}$. The last approximation follows from replacing the exponential by the first two terms of its power series expansion, with the assumption that $\Delta R_{2}^{*}\ll 1$. The optimal BOLD contrast is achieved by setting $T_{E}=T_{2}^{*}$:

(2) $$\begin{array}{l} \Delta S∕S\approx \Delta R_{2}^{*}∕R_{2}^{*} \end{array}$$

The biological response of the tissue drives the change in $\Delta R_{2}^{*}∕R_{2}^{*}$ (Wald, 2012). The choice of 3D EPI or 2D EPI acquisition will not affect the biological response of the tissue, and consequently Δ*S*∕*S* will also be expected to be similar for the two sequences due to the approximate equality with $\Delta R_{2}^{*}∕R_{2}^{*}$. As the typical fMRI analysis scales the entire 4D dataset to compensate for differences in the base signal, *S*, the expectation value for the estimated effect size will be similar for 2D EPI and 3D EPI datasets.

If we assume that the signal has two components, one originating from the task related BOLD response, *S*_task_, and one originating from some physiological fluctuation, *S*_phys_, the temporal signal is:

(3) $$\begin{array}{l} S\left(t\right)=S_{\text{task}}\left(t\right)+S_{\text{phys}}\left(t\right) \end{array}$$

Assuming that *S*_phys_ is proportional to the fluctuation in the physiological parameter, *p*, we get:

(4) $$\begin{array}{l} S_{\text{phys}}\left(t\right)=c\cdot p\left(t\right) \end{array}$$

Here the constant, *c*, will describe the sensitivity to the physiological parameter for the given acquisition method. This could be different for 2D EPI and 3D EPI. The signal is typically scaled by the mean value of the 4D dataset, $\bar{S_{\text{4D}}}$, and demeaned:

(5) $$\begin{array}{l} f\left(t\right)=\frac{S\left(t\right)-\bar{S\left(t\right)}}{\bar{S_{\text{4D}}}}=f_{\text{task}}\left(t\right)+f_{\text{phys}}\left(t\right) \end{array}$$

The physiological signal fluctuation can be decomposed in a component which is correlated with the task, $f_{\text{phys}}^{∥}\left(t\right)$, and a component which is uncorrelated with the task, $f_{\text{phys}}^{⊥}\left(t\right)$:

(6) $$\begin{array}{l} \begin{array}{cc} f\left(t\right) & =f_{\text{task}}\left(t\right)+f_{\text{phys}}^{∥}\left(t\right)+f_{\text{phys}}^{⊥}\left(t\right)\\ =f_{\text{task}}\left(t\right)\left[1+k\right]+f_{\text{phys}}^{⊥}\left(t\right) \end{array} \end{array}$$

Here *k* describes the magnitude of the task-correlated physiological fluctuation relative to the true task induced signal time course.

A traditional general linear model (GLM) in the fMRI, without nuisance regressors, would only attempt to model the signal fluctuation due to the task, i.e., *f*_task_(*t*):

(7) $$\begin{array}{l} Y\left(t\right)=\beta_{\text{task}}^{†}X_{\text{task}}\left(t\right)+\varepsilon^{†}\left(t\right),\;\varepsilon^{†}\left(t\right)\sim N\;\left(0,\sigma^{2}\right) \end{array}$$

The regressor *X*_task_(*t*) is assumed to be a valid description of *f*_task_(*t*) and the least squares estimation of $\beta_{\text{task}}^{†}$ will be biased by the factor *k* in Equation (6), since $f_{\text{phys}}^{∥}\left(t\right)$ cannot be distinguished from *f*_task_(*t*). Uncorrelated physiological signal changes will not introduce any bias in $\beta_{\text{task}}^{†}$, but will be incorporated in the estimation of the errors, ε^†^.

Nuisance regressors can be introduced in the GLM to better account for the physiological signal contribution:

(8) $$\begin{array}{rcl} Y\left(t\right) & = & \beta_{\text{task}}X_{\text{task}}\left(t\right)+\beta_{\text{phys}}X_{\text{phys}}\left(t\right)+\varepsilon \left(t\right),\\ \varepsilon \left(t\right)\sim N\;\left(0,\sigma^{2}\right) \end{array}$$

Where *X*_task_(*t*) is the expected signal time course of the task induced BOLD signal and *X*_phys_(*t*) is the expected signal time course derived from the physiological parameter. To better understand this model, we can decompose the nuisance regressor in components which are correlated and uncorrelated with the task regressor:

(9) $$\begin{array}{l} \begin{array}{cc} Y\left(t\right) & =\beta_{\text{task}}X_{\text{task}}\left(t\right)+\beta_{\text{phys}}\left[X_{\text{phys}}^{∥}\left(t\right)+X_{\text{phys}}^{⊥}\left(t\right)\right]+\varepsilon \left(t\right)\\ =\beta_{\text{task}}X_{\text{task}}\left(t\right)+\beta_{\text{phys}}\left[\xi X_{\text{task}}\left(t\right)+X_{\text{phys}}^{⊥}\left(t\right)\right]+\varepsilon \left(t\right)\\ =\left[\beta_{\text{task}}+\xi \beta_{\text{phys}}\right]X_{\text{task}}\left(t\right)+\beta_{\text{phys}}X_{\text{phys}}^{⊥}\left(t\right)+\varepsilon \left(t\right) \end{array} \end{array}$$

If the expected task related BOLD fluctuations and the physiological signal fluctuations are fully correlated, $X_{\text{phys}}^{⊥}\left(t\right)=0$, Equation (8) does not have a unique solution. However, if we assume that $\left|X_{\text{phys}}^{⊥}\left(t\right)\right|$ is sufficiently large the contributions of the two signal components *f*_task_(*t*) and *f*_phys_(*t*) should be well separated by the by the least squares solution of Equation (8).

It can be noted that if the model without the nuisance regressor (7) is used for a dataset where the signal in reality includes a $f_{\text{phys}}^{∥}\left(t\right)$ component, the physiological fluctuations will affect the parameter estimate and the error estimate in the following two ways. Firstly, the beta estimate will be biased by the task-correlated physiological fluctuations, Equations (7) and (8) gives:

(10) $$\begin{array}{r} \beta_{\text{task}}^{†}=\beta_{\text{task}}+\xi \beta_{\text{phys}} \end{array}$$

Secondly, the error estimate, ε^†^, will be inflated in Equation (7) because it will describe all the variance explained by $\beta_{\text{phys}}X_{\text{phys}}^{⊥}\left(t\right)$ in Equation (8), in addition to the normal contributions to ε (thermal noise, etc):

(11) $$\begin{array}{l} \varepsilon^{†}\left(t\right)=\beta_{\text{phys}}X_{\text{phys}}^{⊥}\left(t\right)+\varepsilon \left(t\right) \end{array}$$

## 3. Materials and methods

### 3.1. Subjects

Thirty-one male healthy volunteers (18–27 years, mean 21 years) participated in the study after giving written informed consent. The study was approved by the National Committee for Medical Research Ethics in Midt-Norge, Norway.

### 3.2. Equipment

The MRI scans were acquired with a Magnetom Trio 3T scanner (*Group A*, *n* = 24) and Magnetom Skyra 3T scanner (*Group B*, *n* = 7) with a Siemens 32 channel head coil (Siemens Healthcare, Erlangen, Germany). The functional tasks were presented to the subject on a monitor positioned at the end of the bore. A joystick with two buttons was used by the subject to control movement within the environments and give responses.

### 3.3. Scanning procedure

Each subject was scanned in two sessions separated by (20 ± 14) days (mean ± sd) in *Group A* and (11 ± 3) days in *Group B*. The difference between these sessions was that 2D EPI was used to acquire the functional scans in one session, while 3D EPI was used in the other session. The order of the sessions was randomized across subjects. For each session one of three different versions of the functional paradigm was used in randomized order. These versions of the functional paradigm differed in the environments the subject should learn, as described below.

Before scanning commenced on the first day, the subjects familiarized themselves with the presentation equipment and joystick and completed practice trials from the different experimental conditions.

#### 3.3.1. Paradigm

**Figure 1:**
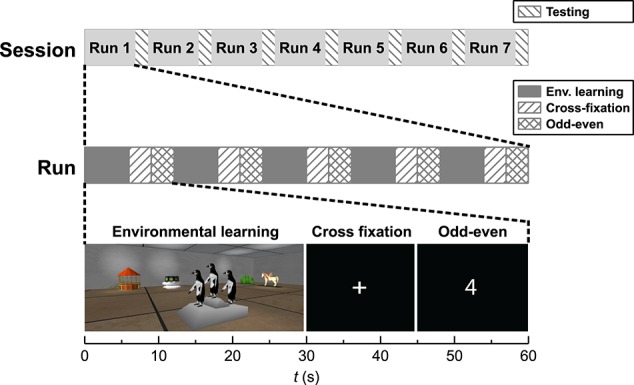
**Schematic view of the functional paradigm**.

An overview of the fMRI scanning procedure is included in Figure 1. The functional paradigm had a block design comprised of three conditions; *environmental learning* (30 s), *cross fixation* (15 s) and *odd*–*even* (15 s). In condition *environmental learning*, the subjects explored a new virtual environment freely from a first person perspective. The environment was comprised of a room with a specific geometric shape and five unique objects placed in the room. The subjects were instructed to learn the geometry of the room, as well as the appearance and position of the objects in the room. In the *cross fixation* condition, the subjects were instructed to further concentrate on memorizing the preceding environment, while a cross on a black background was present on the screen. In the *odd*–*even* condition, random numbers appeared on the screen and the subjects should report on their parity by pressing the left button for odd numbers and right button for even numbers. Five different environments were presented in succession, constituting a functional run. After each run the subject's environmental knowledge was tested. During each session in the scanner the subject completed seven functional runs. The order of the five environments in each run, as well as the order of the runs within the session, was randomized. More details about the study design can be found in Evensmoen et al. (2015).

#### 3.3.2. MRI acquisition

The functional data was acquired with either a 2D EPI sequence or a 3D EPI sequence. The sequences were adapted from the vendor's 2D EPI sequence in the Siemens IDEA framework. Both sequences used the standard navigator based phase correction. The same sequence type was used for all the functional scans within each session. The sequence parameters were set to give similar imaging properties with the different sequences. In *Group A* the 2D sequence used a matrix of 116 × 116, field of view of 220 × 220 mm and slice thickness of 1.9 mm (no gap), with resulting voxel size of 1.9 × 1.9 × 1.9 mm. The volume was imaged by 40 slices in interleaved acquisition. Similarly, the 3D sequence used a matrix of 116 × 116 × 44, field of view of 220 × 220 × 83.6 mm, with resulting voxel size of 1.9 × 1.9 × 1.9 mm. The 2D EPI had *T*_E_ = 28 ms, *T*_R_ = 2110.8 ms, *T*_R_vol__ = 2.111 s and 90° flip angle. The 3D EPI had *T*_E_ = 28 ms, *T*_R_ = 53.7 ms, *T*_R_vol__ = 2.365 s and 16° flip angle. Both sequences had a bandwidth per pixel of 2156 Hz, GRAPPA acceleration by a factor four and the same fat saturation. In the 3D EPI an entire plane in *k*-space was acquired following a single radiofrequency excitation pulse for each partition encoding. The 3D EPI sequence was gradient and radiofrequency spoiled. The imaging slab was obliquely angled at approximately 45° from transversal toward coronal and positioned so that it covered the hippocampi with slices close to perpendicular to the long axis of the hippocampi. For *Group B* the scanning parameters were identical to the ones used for *Group A*, except for repetition times, slice thickness and bandwidth. The 2D EPI had *T*_R_ = 2253.2 ms, *T*_R_vol__ = 2.253 s and slice thickness of 2.0 mm (no gap). The 3D EPI had *T*_R_ = 57.3 ms and *T*_R_vol__ = 2.523 s. The bandwidth per pixel was 1306 Hz for both sequences.

An anatomical *T*_1_ weighted image was also acquired in each session using a 3D MPRAGE. Matrix size: 256 × 256 × 192, 1 mm isotropic resolution, *T*_I_ = 1100 ms, *T*_E_ = 2.96 ms, *T*_R_ = 2300 ms, flip angle: 8° and GRAPPA acceleration by a factor two.

#### 3.3.3. Physiological measurements

For *Group B*, the subjects' heart rates were monitored by recording an electrocardiogram (ECG) during the functional scans using the MR system vendor's equipment.The subjects' respiration was monitored with a sensor belt placed around the lower chest and recorded using a PowerLab 4/30 acquisition device (ADInstruments) started by a trigger input.

### 3.4. Analysis

All analysis was performed using MATLAB (MathWorks, Natick, Massachusetts, U.S.A.) unless stated otherwise.

#### 3.4.1. Physiological measurements

Time series of the subject's heart beats were extracted from the ECG data, using a semi-automated procedure. For each session one channel was selected. Then a distinct signal feature in the heart interval was selected manually, within a part of the recording following the end of the scan (thus free of gradient noise), and averaged across the runs. A wavelet was generated from the averaged selection. Then the coefficient of a continuous wavelet transform of the ECG signal, using the generated wavelet at the original scale, was calculated. The heart beats in the ECG signal were identified by setting the heart beats on the rising edge of a threshold for this coefficient of the continous wavelet transform. Finally the selected heartbeats were inspected together with the ECG signal and errors were manually corrected.

The raw respiration data were low pass filtered using a second order Butterworth filter with passband up to 0.45 Hz.

Nuisance regressors based on the physiological measurements were created using FSLs Physiological Noise Modeling (Analysis Group, FMRIB, Oxford, UK) (Brooks et al., 2008). Using the heart beat time series and the filtered respiration signal as input ten nuisance regressors were created: eight RETROICOR (Glover et al., 2000) type regressors (2nd order cardiac and 2nd order respiratory), as well as one for cardiac rate and one for respiratory volume per time (RVT) (Birn et al., 2006). The software default 10 s smoothing was applied on the cardiac rate and RVT regressors.

#### 3.4.2. FMRI analysis

The images were analyzed using FSL 5.0.1 (Analysis Group, FMRIB, Oxford, UK). Non-brain tissue was removed from the anatomical *T*_1_ weighted image using FSL's BET. Then the anatomical brain image was co-registered to the MNI 152 standard space (Montreal Neurological Institute, Montreal, QC, Canada) by a non-linear transform, using FSL's FNIRT. For the functional data acquired with the 3D sequence, the two outer reconstructed slices at either end of the imaged slab were removed. Each functional run was motion corrected using FSL's MCFLIRT followed by non-brain tissue removal using FSL's BET. Thereafter, the functional run was co-registered to the anatomical *T*_1_ weighted brain image by a rigid body transform using FSL's FLIRT with a normalized mutual information cost function. Further pre-processing included spatial smoothing with a 3 mm full width at half maximum Gaussian kernel and temporal high pass filtering with a 100 s cut off period. Activation statistics were calculated using FSL's FEAT. The conditions were modeled using a box car function convolved with a gamma hemodynamic response function. Two contrasts were defined:

- ***Learning***: condition *environmental learning* − condition *odd*–*even*
- ***Crossfixation***: condition *cross fixation* − condition *odd*–*even*

The calculation of the activation statistics was repeated using four different GLM-models. *Model 1*: Included the functional conditions and temporal derivatives. *Model 2*: Included six nuisance regressors generated from the motion correction parameters (option in FEAT) in addition to regressors included in *Model 1*. *Model 3*: Included nuisance regressors generated from the cardiac activity and respiration using Physiological Noise Modeling as described above in addition to *Model 1*. *Model 4*: Included all the nuisance regressors included in *Model 2* and *Model 3* in addition to *Model 1*. The models used are summarized in Table 1. First level statistics were calculated for each run. Thereafter, session level statistics (within subject) were calculated using a fixed effects model. Finally, group level statistics for all sessions acquired with each sequence type were calculated using a mixed effects model (FLAME 1).

**Table 1:** **Summary of regressors included in the different models**.

**Regressors**	**Number of regressors**	**Model**			
		**1**	**2**	**3**	**4**
Functional task conditions and temporal derivatives	6	×	×	×	×
Motion correction parameters (rotations and translations)	6		×		×
Cardiac up to 2nd order	4			×	×
Respiratory up to 2nd order	4			×	×
Cardiac rate	1			×	×
Respiratory volume per time	1			×	×

The design matrix for *Model 4* was used to calculate the correlation coefficient of the different nuisance regressors and the functional contrasts for each run. Thereafter, the mean correlation coefficient was estimated for each nuisance regressor, as well as the confidence interval for the mean correlation coefficient.

#### 3.4.3. Effect of model

From each individual run the mean value of the contrast estimates was calculated. A set of analysis-of-variance (ANOVA) was carried out using the mean of the contrast estimates as the response variable. *Subject*, *Sequence* (2D/3D), *Day* (first- or second-session), and *Run* were used as factors. Separate ANOVAs were conducted for each contrast and for each model. In total eight separate ANOVAs were performed in *Group B* and four separate ANOVAs in *Group A*. Only voxels that were included in all runs for all subjects were included in the calculation of the response variable. This was done to avoid that variance associated with e.g., positioning of the imaging slab, which was random with respect to all of the included factors, affected the analysis.

To further investigate the effect of the choice of model, a joint histogram of the contrast estimates for *Model 1* and *Model 4* on group level voxel basis was made. A separate histogram was made for each contrast and for both the 2D and 3D EPI datasets. For each histogram a fitted straight line was estimated by a straight line fitting to data with errors in both coordinates (Press et al., 2007).

In all statistical analysis, a significance level of *p* < 0.05 was used.

To evaluate the quality of the models the Akaike's information criterion, AIC (Akaike, 1973; Burnham and Anderson, 2003), was also calculated for each model. We used a corrected version of the AIC, to account for a small sample size, AIC_*c*_, defined as:

(12) $$\begin{array}{l} {\text{AIC}}_{c}=\text{AIC}+\frac{2k\left(k+1\right)}{n-k-1} \end{array}$$

(13) $$\begin{array}{l} \text{AIC}=n\ln\left(\frac{\text{RSS}}{n}\right)+2k \end{array}$$

where RSS is the residual sum of squares of the regression, *n* is the number samples and *k* is the number of estimated parameters. In this formulation a constant term dependent on the specific dataset is left out, and comparisons will only be valid for comparing different models applied on the same dataset. The Akaike's information criterion gives a measure of the goodness of fit of the model relative to the complexity of the model, a lower value indicates the better model. In addition to the number of regressors, *k* was increased by 2 to account for the variance estimate and the intercept (demeaning). The mean RSS value within a mask was calculated for each run, only voxels which were included in all runs for all subjects were included in the mask. Thereafter, the AIC_*c*_ value for each run was calculated using Equation 12 and finally the mean AIC_*c*_ for the group was calculated for each model.

As a measure of the overlap of the statistical maps generated from the 2D EPI and 3D EPI datasets, correlation coefficients for group contrast estimates were calculated. The correlation coefficient for the contrast estimates was calculated using the group level estimates for all voxels included in both datasets.

## 4. Results

**Figure 2:**
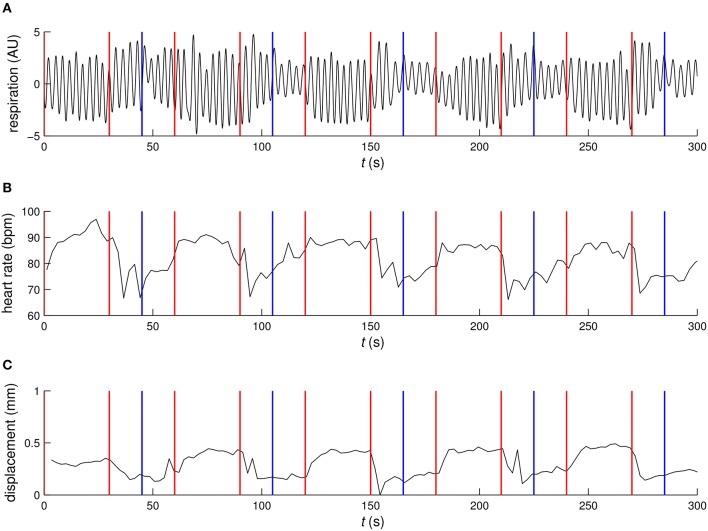
**Example time courses of (A) respiration, (B) heart rate, and (C) motion**. Red vertical lines mark the start and end of the environmental learning condition and blue vertical lines mark the transition form the cross fixation condition to the odd–even judgment condition.

**Figure 3:**
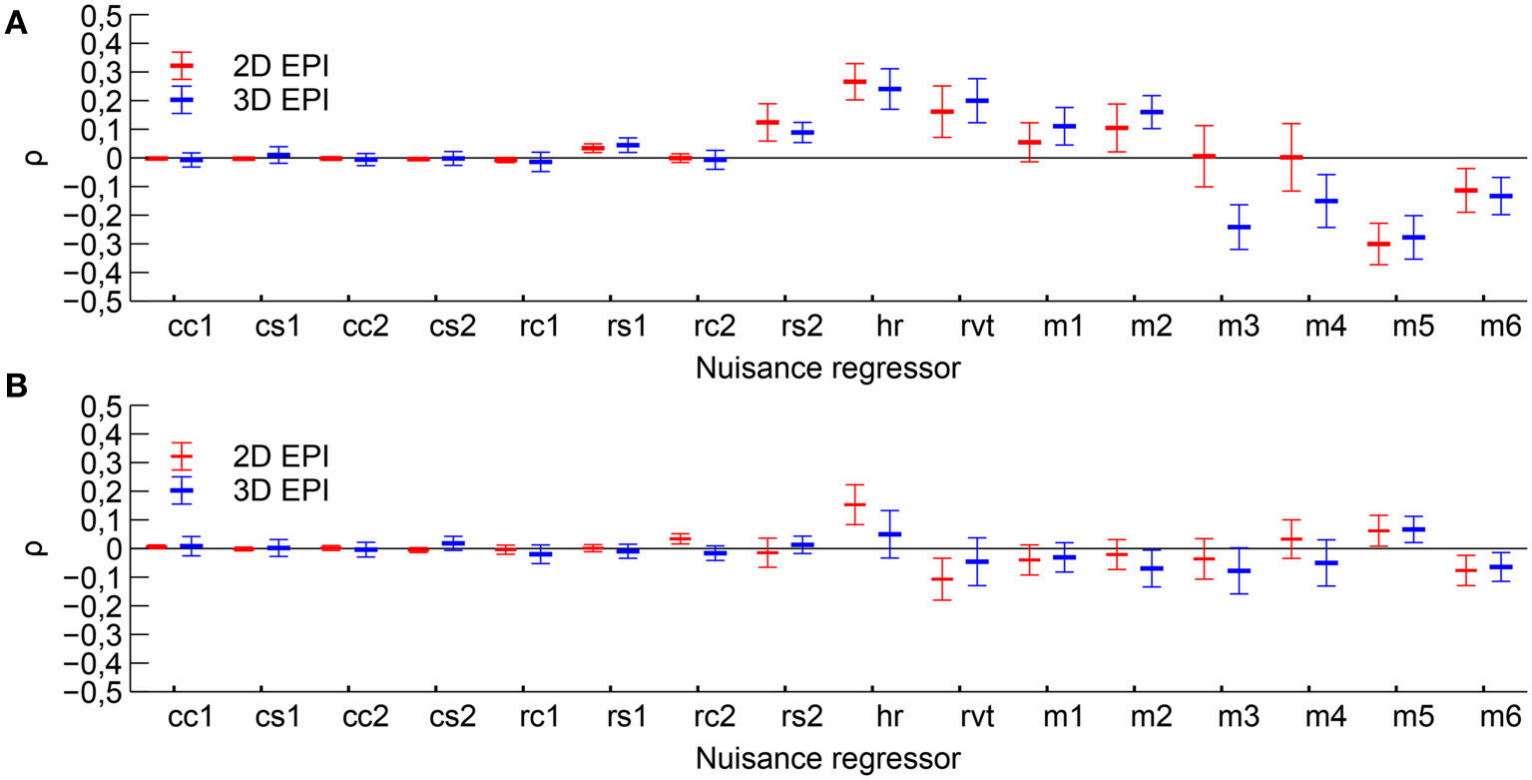
**Mean correlation coefficients between the nuisance regressors and time course of the expected BOLD signal change for each contrast. (A)** Correlation with contrast Learning and **(B)** correlation with contrast Cross fixation. Mean value and 95 % confidence interval is indicated. Nuisance regressors *cc*1–*cs*2 are the RETROICOR type cardiac regressors, *rc*1–*rs*2 are the RETROICOR type respiratory regressors, *hr* is the heart rate regressor, *rvt* is the respiratory volume per time regressor and *m*1–*m*6 are the motion regressors.

The example time courses for physiological parameters and motion, see Figure 2, showed clear signs of correlation with functional tasks. Quantitatively, the RVT regressor, heart rate, several of the motion regressors and some of the respiratory phase based regressors showed partial correlation with the *Learning* contrast, see Figure 3. For the *Cross fixation* contrast the correlation with nuisance regressors was smaller. The correlation results for 2D EPI and 3D EPI had overlapping confidence intervals except for two cases, these were the motion regressor *m*3 correlated with the *Learning* contrast and the respiratory phase regressor *rc*2 correlated with the *Cross fixation* contrast.

The results from the ANOVA analyses are summarized in Tables 2, 3. For *Group A*, where only *Model 1* and *Model 2* were applied, *Sequence* was a significant factor only for the contrast *Learning*. For *Group B Sequence* was a significant factor for *Model 1*–*3* for the *Learning* contrast and only for *Model 1* for the *Cross fixation* contrast. Using *Model 4*, *Sequence* was not a significant factor in any of the contrasts.

**Table 2:** ***Group A p*-values for analysis-of-variance using the mean of the contrast estimates per run as response variable**.

**Model**	**Learning**				**Cross fixation**			
	**Subject**	**Sequence**	**Day**	**Run**	**Subject**	**Sequence**	**Day**	**Run**
1	< 0.001^*^	< 0.001^*^	0.420	0.012^*^	< 0.001^*^	0.357	0.892	0.717
2	< 0.001^*^	< 0.001^*^	0.421	0.662	< 0.001^*^	0.496	0.782	0.906

* *Statistically significant factors*.

**Table 3:** ***Group B p*-values for analysis-of-variance using the mean of the contrast estimates per run as response variable**.

**Model**	**Learning**				**Cross fixation**			
	**Subject**	**Sequence**	**Day**	**Run**	**Subject**	**Sequence**	**Day**	**Run**
1	0.001^*^	< 0.001^*^	0.027^*^	0.116	< 0.001^*^	0.011^*^	0.745	0.422
2	0.001^*^	0.049^*^	0.555	0.269	0.021^*^	0.114	0.643	0.326
3	0.001^*^	0.010^*^	0.117	0.097	< 0.001^*^	0.239	0.474	0.307
4	0.006^*^	0.271	0.750	0.252	0.002^*^	0.379	0.808	0.164

* *Statistically significant factors*.

**Figure 4:**
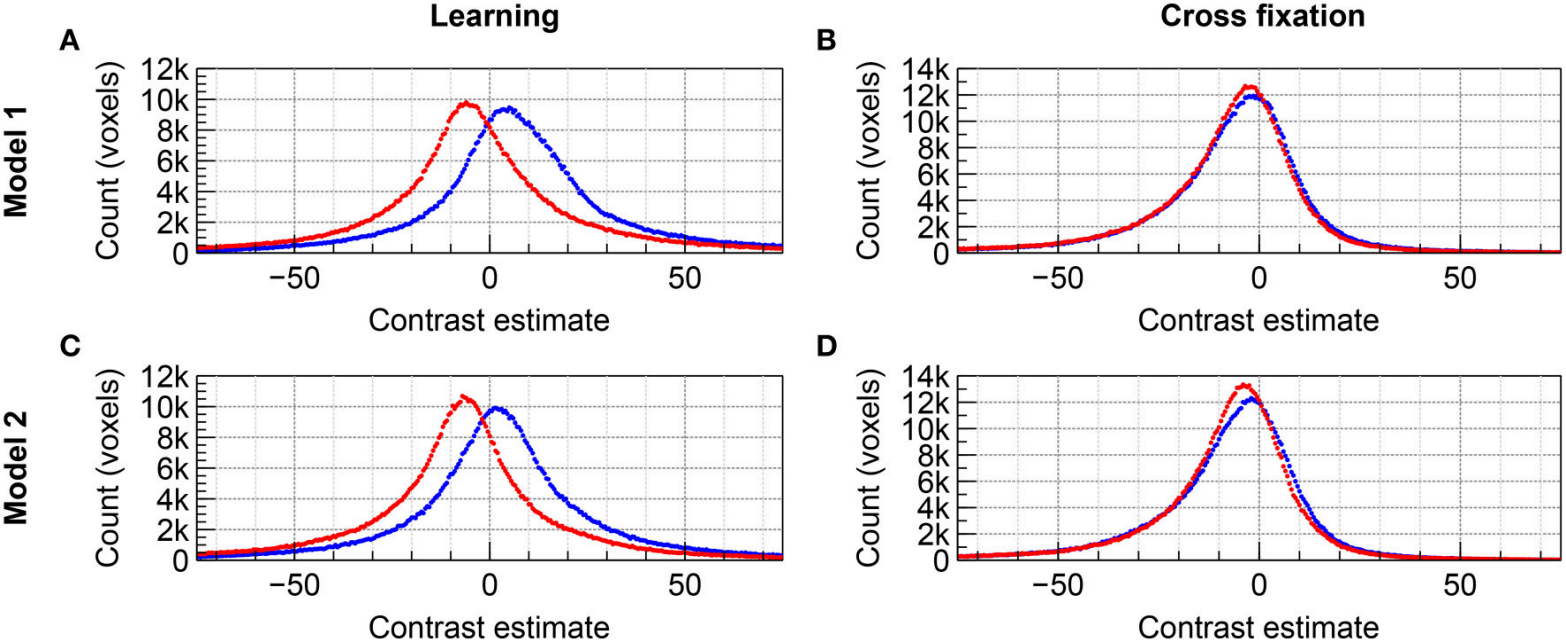
**Comparison of the distribution of contrast estimates for Group A on group level for the datasets acquired with 2D EPI (red) and 3D EPI (blue)**. Using *Model 1*, **(A)** contrast *Learning* and **(B)** contrast *Cross fixation*. Using *Model 2*, **(C)** contrast *Learning* and **(D)** contrast *Cross fixation*. The histogram bin size was 0.5.

**Figure 5:**
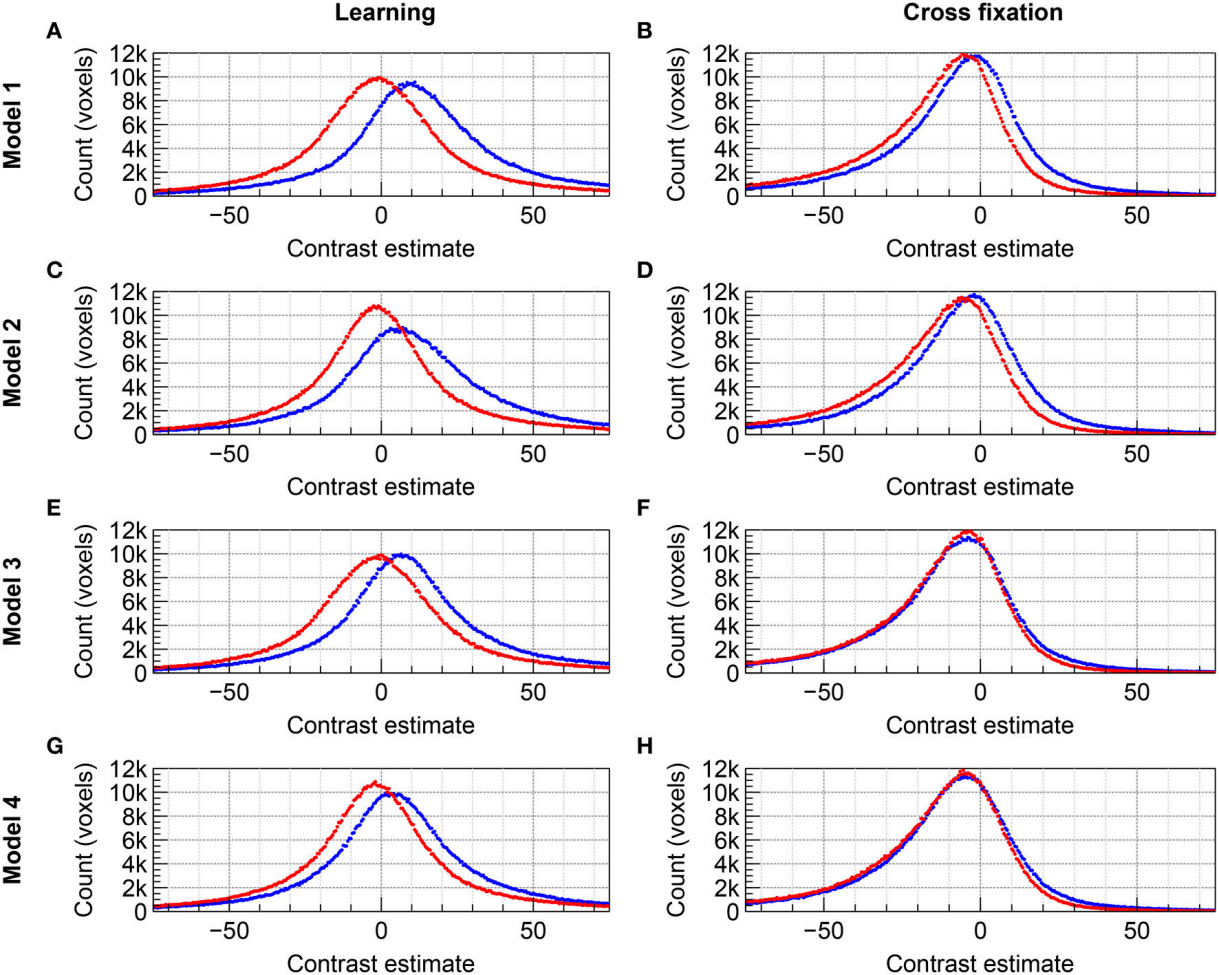
**Comparison of the distribution of contrast estimates for Group B on group level for the datasets acquired with 2D EPI (red) and 3D EPI (blue). (A,B)** *Model 1*, **(C,D)** *Model 2*, **(E,F)** *Model 3*, and **(G,H)** *Model 4*. **(A,C,E,G)** contrast *Learning*. **(B,D,F,H)** contrast *Cross fixation*. The histogram bin size was 0.5.

The distributions of group level contrast estimates for the data acquired by imaging with 2D EPI and 3D EPI are shown in the histograms included in Figure 4 for *Group A* and Figure 5 for *Group B*. Using *Model 1*, a large difference in the distributions was seen for contrast *Learning* for both groups, the distributions appeared shifted relative to each other. For contrast *Cross fixation*, the distributions had a high degree of overlap for *Group A*, while a small shift was visible for *Group B*. Using *Model 2*, a change toward more overlapping distributions, compared to *Model 1*, was seen for contrast *Learning* in both groups of subjects. No clear change compared to *Model 1* was visible for contrast *Cross fixation*. Using *Model 3* (*Group B* only), the distributions were changed compared to *Model 1* for both contrasts, again toward more overlap between the contrast estimates based on the 2D EPI and 3D EPI datasets. When using *Model 4* (*Group B* only), the difference between the distributions was further reduced compared to *Model 1*–*3* for contrast *Learning*.

**Figure 6:**
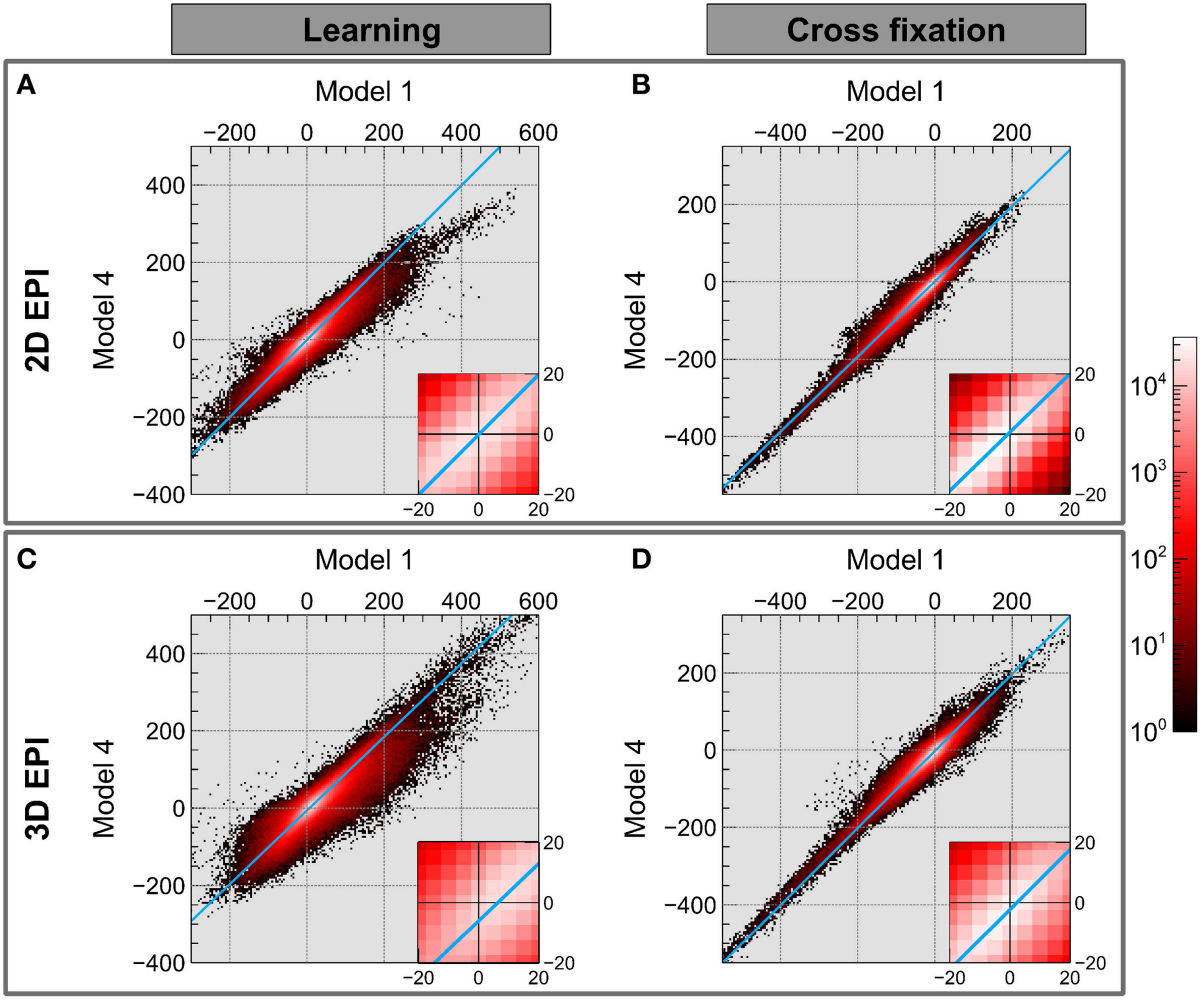
**The joint histogram of the contrast estimates for Model 1 and Model 4 on group level voxel basis is shown in on a logarithmic scale (black-red-white)**. Using the 2D EPI data: **(A)** contrast *Learning* and **(B)** contrast *Cross fixation*. Using the 3D EPI data: **(C)** contrast *Learning* and **(D)** contrast *Cross fixation*. The light blue line shows a straight line fitting to data with errors in both coordinates.

The results of comparing the group level contrast estimates using *Model 1* and *Model 4* for each voxel are shown in the joint histograms in Figure 6. It was observed that choice of model has a larger effect on contrast estimates for contrast *Learning* than for contrast *Cross fixation* also when examining each voxel. The straight line fit to the data intersects the axis close to the origin for both contrasts using the 2D EPI datasets. When using the 3D EPI datasets the intersection was at an offset from the origin, indicating a general shift in contrast estimate values between **Model 1** and **Model 4**.

**Figure 7:**
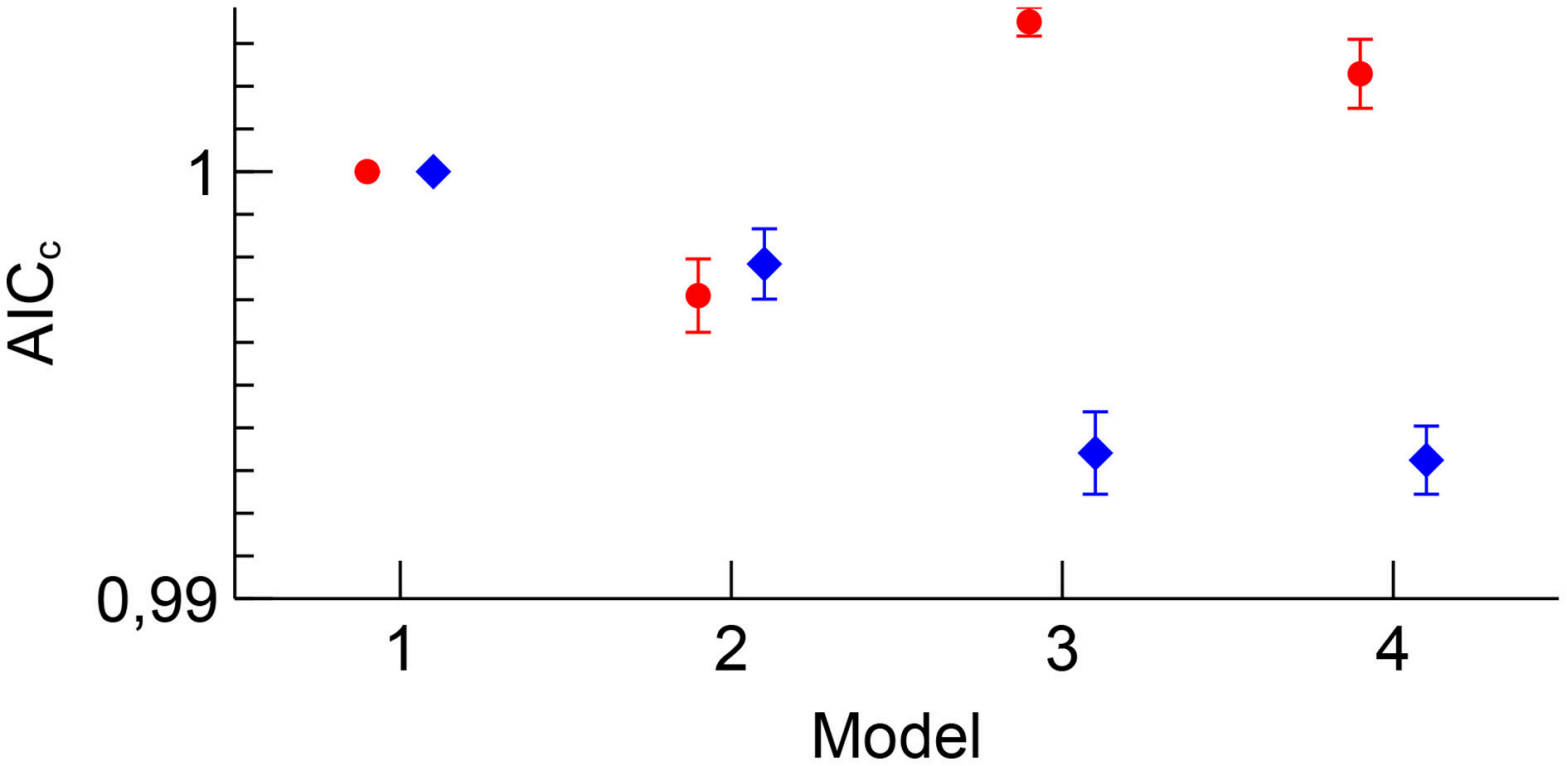
**Group B mean AIC_*c*_ for 2D EPI (red) and 3D EPI (blue) for each model**. Errorbars show the standard error of the mean. The values have been normalized to the value for Model 1 for each sequence.

The AIC_*c*_ quality of fit for each of the four models is reported in Figure 7. While the 3D dataset benefited from adding more nuisance regressors, the 2D EPI dataset only benefited from adding motion parameters, indicating that the physiological fluctuations did not affect the 2D EPI data as much as the 3D EPI data.

The calculated correlation coefficients between the contrast estimate maps estimated from the 2D EPI and the 3D EPI datasets, are listed in Table 4. A high correlation, between the contrast estimates in the 2D EPI and 3D EPI dataset, was observed for both *Learning* and *Cross fixation* for both groups.

**Table 4:** **Correlation between group level contrast estimates for the 2D EPI and 3D EPI datasets**.

**Model**	**Group A**		**Group B**	
	**Learning**	**Cross fixation**	**Learning**	**Cross fixation**
1	0.91	0.90	0.78	0.85
2	0.91	0.88	0.78	0.83
3			0.78	0.84
4			0.80	0.83

**Figure 8:**
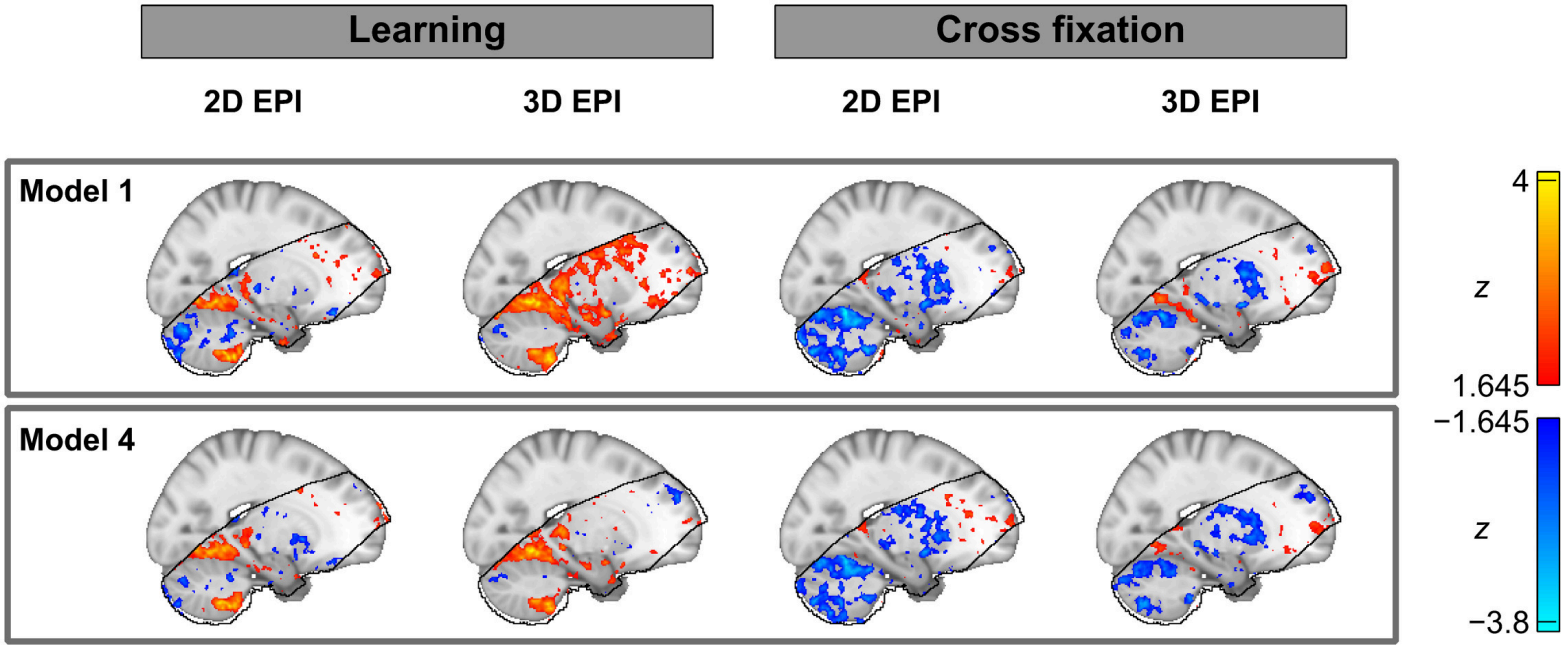
**Thresholded group level activation maps (*z*-values) overlaid on an example slice of the MNI152 1 mm standard template**. The black line outlines the volume included in the group analysis of both the datasets acquired by 2D EPI and 3D EPI.

Finally, thresholded activation statistics maps (*z*-values) overlaid on a saggital example slice of the MNI152 1 mm standard template are included in Figure 8. Although this map will show a mix of both voxels which have no true activation and voxels which have true activation, it has been included to give the reader an impression of how the bias of the contrast estimates affects the *z*-statistics. The extent of the fMRI analysis mask is also indicated.

## 5. Discussion

This study was based on previous reports in the literature which have found that (1) in higher cognitive level paradigms, physiological parameters like heart rate and respiration vary compared to baseline or simple task paradigms, and that (2) 3D EPI is more sensitive to physiological fluctuations than 2D EPI. The combination of the above two effects would be that 2D EPI is less influenced by partly task-correlated physiological fluctuations than 3D EPI.

### 5.1. Task-correlated physiological fluctuations and motion

Two different contrasts were analyzed in this study, *Learning* and *Cross fixation*. The *Learning* contrast was taken between the *environmental learning* condition, which involved active navigation using a joystick combined with scene exploration (active engagement) and memory encoding (a high-level cognitive task); and the *odd*–*even* condition, which involved odd-even number recognition (a less demanding cognitive task) in combination with a button press. So for the *Learning* contrast there was a clear difference in cognitive level and in addition an expected difference in involuntary head motion from the joystick usage and active navigation. The *Cross fixation* contrast was taken between the *cross fixation* condition; which involved memorizing the navigated environment (a high-level cognitive task), but without joystick usage and active navigation; and the *odd*–*even* condition. So for the *Cross fixation* contrast there was still a difference in cognitive level, but less expected difference in involuntary head motion.

As discussed in the introduction, increased heart rate and respiratory volume per time have been observed during the active periods in higher cognitive task paradigms (Backs and Seljos, 1994; Veltman and Gaillard, 1998) and with greater working memory load (Backs and Seljos, 1994; Gianaros et al., 2004; Mehler et al., 2009). For simple paradigms such increases in heart rate or respiration do not occur (Conrad and Klingelhöfer, 1989). One would therefore expect task-correlated physiological fluctuations in both the *Learning* contrast and the *Cross fixation* contrast, but larger task-correlated motion in the *Learning* contrast. The results, shown in Figure 3, were consistent with these predictions, showing an overall higher correlation for the *Learning* contrast, in particular for the motion parameters.

In general, the correlation of the nuisance regressors with the contrasts was similar for 3D EPI and 2D EPI acquisition, indicating that the dataset from *Group B* was sufficiently large to even out most of the individual variations in physiological state between test and retest. However, for *z*-translation (*m*3) in the *Learning* contrast and respiratory phase (*rs*2) in the *Cross fixation* contrast, the confidence intervals did not overlap. Since one in general does not expect the physiological state to depend on the details of the MRI acquisition method, this was a surprising result. One possible explanation was the small sample size, but for the motion-related parameter there was an additional possible explanation. The motion parameters were extracted from the motion correction of the MRI data. Head motion within the time frame of the acquisition of a single volume will propagate differently into the images for 3D EPI vs. 2D EPI. This may result in different motion correction results even if the actual head motion was the same. Without externally acquired motion data it is not possible to conclude on this issue.

### 5.2. Task-correlated signal fluctuations

From Equation (10) it follows that when task-correlated physiological fluctuations and/or motion are present, the contrast estimate will be shifted away from its true (intrinsic) value. In addition, if 2D EPI and 3D EPI have different sensitivity to physiological fluctuations and motion [the constant *c* in Equation (4)], the bias of the contrast estimate will differ between the two sequences. Since we do not know the true/intrinsic contrast value, we cannot directly determine the size of the bias. However, we can observe such a bias indirectly if the sensitivity factor *c* differs between sequences. In Figure 4 for *Group A* and Figure 5 for *Group B* there was a very distinct difference in the group level contrast estimate distributions between 2D EPI and 3D EPI for the *Learning* contrast, while very little such difference was seen for the *Cross fixation* contrast. Held together with the observed difference in task-correlated physiological fluctuations and motion between the two contrasts (as discussed above), we interpret this as a clear indication that the signal in 3D EPI and 2D EPI have different sensitivity to physiological fluctuations and motion.

Whether 3D EPI is more or less sensitive than 2D EPI cannot be determined from Figures 4, 5 alone. This will be discussed in more detail in the next section.

### 5.3. Effect of including nuisance regressors in fMRI analysis

According to Equation (9), inclusion of nuisance regressors for physiological fluctuation and motion should remove the bias in the contrast estimates, and thus also any systematic bias between contrast estimates in 2D EPI and 3D EPI.

The ANOVA analysis of the mean contrast estimates revealed that when including the full set of nuisance regressors (*Model 4*), *Sequence* was no longer a significant factor. However, the distribution of group level contrast estimates (Figure 5) was not showing quite the same result. Here there was a residual bias left between 2D EPI and 3D EPI in the *Learning* contrast, even for *Model 4*. Held together, nuisance regressors did reduce the difference between 2D EPI and 3D EPI significantly, but did not necessarily remove it completely.

Figure 6 shows that for the *Learning* contrast and 3D EPI sequence, the contrast estimates were shifted between *Model 1* and *Model 4*, indicated by the blue line not crossing through the origin. For 2D EPI there was not a systematic shift in the contrast estimates (the blue line crosses through the origin). For the *Cross fixation* contrast, this effect was smaller, but still present.

A second effect of adding appropriate nuisance regressors to the model is that the residuals should be reduced, because unmodeled fluctuations which are uncorrelated to the paradigm will be added to the residuals, as shown by Equation (11). The Akaike's information criterion was used to assess whether the added number of regressors in a model including nuisance regressors was justified by a sufficiently large reduction in the residuals. The analysis revealed an interesting difference between 2D EPI and 3D EPI. For 2D EPI the *AIC*_*c*_ increased when nuisance regressors for cardiorespiratory fluctuations were included (*Model 3*), indication that the residuals were not much affected by these physiological fluctuations. On the contrary, the *AIC*_*c*_ value for the 3D EPI dataset showed that this set of nuisance regressors was very important and that the residuals were reduced. The amount of signal from physiological fluctuations which affects the *AIC*_*c*_ is determined by the magnitude or amount of the physiological fluctuations in itself, the degree of correlation with the paradigm, and how sensitive the acquisition is to the physiological fluctuations. We assume that the sample size is sufficiently large, and that there is no systematic difference in the amount of physiological fluctuations in the 2D EPI and 3D EPI sessions. Further, the correlation between the nuisance regressors and the contrasts were generally similar (Figure 3). Given these two conditions, the difference in the contribution to the residuals between the 2D EPI and 3D EPI datasets is an indication that the sensitivity to the physiological fluctuations was higher in the 3D EPI acquisition. This result is in line with the findings of several studies investigation temporal noise in 3D EPI, compared to 2D EPI (Goerke et al., 2005; van der Zwaag et al., 2012; Jorge et al., 2013).

Taken together, our results are consistent with 3D EPI having a high sensitivity to physiological fluctuations (i.e., a large value of the sensitivity constant *c*), while 2D EPI has lower sensitivity.

### 5.4. Other effects and limitations

Regardless of which model was used *Subject* was found to be a statistically significant factor in the ANOVAs. Large inter-subject differences in neuronal activation are common (Machielsen et al., 2000; Miller et al., 2002; Wagner et al., 2005; Bennett and Miller, 2010). In this paradigm the subjects' success in performing the task and abilities with the joystick movement, as well as choice of strategy may further add to inter-subject differences (Iaria et al., 2003).

For *Group B* and *Model 1* the factor *Day* was statistically significant for contrast *Learning*. With a group size of 7 it was not possible to make the *Day* factor fully orthogonal to the *Sequence* factor, so they are partially collinear. The fact that *Day* had a low *p*-value only in cases when the *p*-value for *Sequence* was very low for contrast *Learning*, and that *Day* had a high *p*-value in all other ANOVAs, suggests that *Day* may have picked up some variance associated with the *Sequence* factor in the ANOVAs for *Model 1* and *Learning*. In *Group A* with n = 24, *Day* was not a significant factor, while *Sequence* was still a highly significant factor for *Learning*. At this group size, orthogonality between *Day* and *Sequence* is better.

For *Group A Run* was a significant factor using *Model 1*, but not using *Model 2*. For *Group B* no such effect was observed. It is difficult to explain such differences, but since the significance was removed when adding motion regressors to the model, it is natural to assume that the original effect was related to difference in motion. One possible explanation would then be that the degree or pattern of motion change from the start of the experiment toward the end. Maybe the subjects became uneasy or more relaxed over time. Since we did not test for such an effect, this is only a speculation at present.

In this study, the 3D EPI was accelerated by parallel imaging only along the blipped phase encoding direction and not along the second phase encoding (slice) direction. This choice reduces geometric distortion which is a problem in regions in the inferior medial temporal lobe of interest with regard to environmental learning. However, in the general case it might be more favorable to include acceleration along the second phase encoding direction in order to reduce *T*_R_vol__ and consequently the sensitivity to physiological noise (van der Zwaag et al., 2012).

Recent developments in simultaneous multi-slice EPI are also very promising with respect to reduced sensitivity to physiological noise, since it offers the combination of single-shot readout with short *T*_R_vol__ (Poser et al., 2014; Zahneisen et al., 2015).

## 6. Conclusions

The results presented in this study illustrate that the use of 2D EPI or 3D EPI for BOLD fMRI in a higher cognitive level paradigm can have an effect on the contrast estimate results. Our analysis points to the higher sensitivity to physiological fluctuations in 3D EPI as a main mechanism. Hence, the inclusion of nuisance regressors for motion, cardiac- and respiratory phase, heart rate and respiratory volume per time, reduces the bias in the contrast estimates for 3D EPI, thereby reducing the differences between the 2D EPI and the 3D EPI datasets. However, some signal bias between the datasets may remain, and so one cannot guarantee that the expectation value of the contrast estimates from 2D EPI and 3D EPI will be equivalent. If 3D EPI is used for fMRI acquisition in situations in which task-correlated fluctuations in physiological parameters or motion may occur, proper correction of resulting signal fluctuations is essential in order to minimize introduction of bias in the contrast estimates.

## Author contributions

JL programmed the adaptations to the EPI sequences and performed most of the analysis. HE prepared the virtual environmental learning stimulus. JL and HE contributed to the data collection. AH, AK, and PG supervised the project. All authors listed contributed to writing the paper and approved it for publication.

### Conflict of interest statement

The authors declare that the research was conducted in the absence of any commercial or financial relationships that could be construed as a potential conflict of interest.
